# Healthy Brain Aging Education as a Community Engaged Research Strategy With a Tribal Nation

**DOI:** 10.1093/geroni/igaf122.1116

**Published:** 2025-12-31

**Authors:** Mary Wyman, Sarah Punshon, Jenna Woestman, Celina Whitmore, Kala Cornelius, Danielle Lennon, Wesley Martin, Jr., Steve Barczi

**Affiliations:** UW–Madison and Veterans Affairs, Madison, Wisconsin, United States; William S. Middleton Memorial Veterans’ Hospital, Madison, Wisconsin, United States; Veterans Affairs, Madison, Wisconsin, United States; Veterans Affairs, Madison, Wisconsin, United States; University of Wisconsin–Madison, Madison, Wisconsin, United States; Veterans Affairs, Madison, Wisconsin, United States; University of Wisconsin–Madison, Madison, Wisconsin, United States; Veterans Health Administration, Madison, Wisconsin, United States

## Abstract

Due to the effects of longstanding social inequities and historical trauma, Native American (NA) older adults may disproportionately experience barriers to accessing adequate healthcare, home and community-based services, and educational resources to support aging well. Efforts to address these barriers through effective collaborations are critical to support healthy aging and reduce disparities, especially in rural settings. This presentation reports on community education delivered as part of a pilot project to adapt an established telehealth dementia care protocol for implementation in one rural NA community. The project is a collaborative effort between the Tribal healthcare system, Veterans Affairs, and the University of Wisconsin. As a key strategy for community engagement and sustainment of the clinical program, brain health information is provided to older adults and their families, and through a partnership with an intertribal aging organization, wider dissemination within the region is attained. The educational sessions are conducted in-person at larger social events and include presenters both from the Tribal Nation and from external partners. Topics focus on awareness of age-related cognitive changes and lifestyle choices that can maintain or improve brain health, and have included the impacts of brain injury on cognition, healthy lifestyle behaviors that promote aging well, and an overview of available community resources. The authors describe the process of co-designing and co-delivering the educational events with NA community members and report on data from participant surveys demonstrating acceptability and satisfaction.

